# Facilitating drug delivery in the central nervous system by opening the blood-cerebrospinal fluid barrier with a single low energy shockwave pulse

**DOI:** 10.1186/s12987-021-00303-x

**Published:** 2022-01-06

**Authors:** Yi Kung, Kuan-Yu Chen, Wei-Hao Liao, Yi-Hua Hsu, Chueh-Hung Wu, Ming-Yen Hsiao, Abel P.-H. Huang, Wen-Shiang Chen

**Affiliations:** 1 Department of Physical Medicine and Rehabilitation, National Taiwan University Hospital & National Taiwan University College of Medicine, Taipei City, Taiwan; 2 Division of Pulmonology, Department of Internal Medicine, National Taiwan University Hospital and College of Medicine, Taipei City, Taiwan; 3 Department of Surgery, National Taiwan University Hospital, Taipei City, Taiwan; 4 Institute of Biomedical Engineering and Nanomedicine, National Health Research Institutes, Miaoli, Taiwan

**Keywords:** Low-energy extracorporeal focused shockwave pulse, Blood–brain barrier, Blood-cerebrospinal fluid barrier, Central nervous system, Glioblastoma multiforme, Leptomeningeal carcinomatosis

## Abstract

**Background:**

The blood-cerebrospinal fluid (CSF) barrier (BCSFB) is critically important to the pathophysiology of the central nervous system (CNS). However, this barrier prevents the safe transmission of beneficial drugs from the blood to the CSF and thus the spinal cord and brain, limiting their effectiveness in treating a variety of CNS diseases.

**Methods:**

This study demonstrates a method on SD rats for reversible and site-specific opening of the BCSFB via a noninvasive, low-energy focused shockwave (FSW) pulse (energy flux density 0.03 mJ/mm^2^) with SonoVue microbubbles (2 × 10^6^ MBs/kg), posing a low risk of injury.

**Results:**

By opening the BCSFB, the concentrations of certain CNS-impermeable indicators (70 kDa Evans blue and 500 kDa FITC-dextran) and drugs (penicillin G, doxorubicin, and bevacizumab) could be significantly elevated in the CSF around both the brain and the spinal cord. Moreover, glioblastoma model rats treated by doxorubicin with this FSW-induced BCSFB (FSW-BCSFB) opening technique also survived significantly longer than untreated controls.

**Conclusion:**

This is the first study to demonstrate and validate a method for noninvasively and selectively opening the BCSFB to enhance drug delivery into CSF circulation. Potential applications may include treatments for neurodegenerative diseases, CNS infections, brain tumors, and leptomeningeal carcinomatosis.

**Supplementary Information:**

The online version contains supplementary material available at 10.1186/s12987-021-00303-x.

## Introduction

The brain is a unique organ in that is highly protected from the periphery by two major barriers, the blood–brain barrier (BBB) and the blood-cerebrospinal fluid barrier (BCSFB). However, the mechanisms by which the BBB and the BCSFB protect the brain/CSF from pathogens and toxins also block drug delivery [1, 2]. However, BCSFB and BBB are different from their functional and morphological domains. Issues related to how to open the BBB have been widely explored over the past decade. However, research on opening the BCSFB and its importance to CSF circulation and brain disorders is still relatively limited [3].

It is possible to achieve widespread drug delivery to the whole brain by opening the BCSFB instead of the BBB. Pharmacologically regulated BBB opening has been shown to provide a wide drug delivery area and a long delivery window; however, this long window poses a high risk of infection. On the other hand, high-intensity focused ultrasound (HIFU), a method commonly used to open the BBB, can provide a shorter delivery window, reducing the risk of infection but limiting the area to which drugs can be delivered [4].

BCSFB opening addresses the limitations of both pharmacological and HIFU-based BBB opening, providing a short opening period and a wide drug delivery area in the brain or the central nervous system (CNS) in general [5]. Currently, drug delivery to CSF is primarily achieved in five ways: (i) intracerebroventricular (ICV) or intrathecal drug infusion, with direct drug injection/infusion into the CSF [6]; (ii) the use of an anti-transferrin receptor (TfR) antibody (OX-26 anti-rat TfR) to transport medication across the BCSFB into the CSF [7]; (iii) delivery from the olfactory region into the olfactory bulb via trigeminal and olfactory neurons, followed by absorption into the lamina propria and then entry into the CSF [8]; (iv) dosing with John Cunningham virus (JCV); and (v) treatment with drug-loaded biodegradable nanoparticles that enhance the adoptive transfer ability of the BCSFB [9, 10]. However, when used for the delivery of therapeutic drugs, these methods are limited by low efficiency, invasiveness, or the development of immunogenic reactions [11]. Thus, an efficient and noninvasive method to effectively control the permeability of the BCSFB is urgently needed.

Previous research on the use of mechanical waves to transport drugs into the CSF circulation mainly applied focused ultrasound to induce opening of the blood-spinal cord barrier (BSCB) [12]. As its name implies, the BSCB exists in the spinal cord and is functionally and morphologically similar to the BBB [13]. Unfortunately, the use of focused ultrasound to open the BSCB may not only be hampered by the complicated bony structure around, but also may induce spinal cord injury, resulting in permanent motor and sensory disability [14]. If mechanical waves are to be used to open one of the blood–brain barriers, they must be applied to a location to which risk of damage would be minimal and/or would have little adverse effect on patient function. Our focused shockwave pulse (FSW)-BCSFB opening method is applied to the lateral ventricle region, which is considered a non-eloquent and safe region [15]. Furthermore, with our technique, CSF circulates from the lateral ventricle area to the spinal cord area, which is reversed for the BSCB opening technique. In our previous study, we have shown that even at severe epileptic attacks, our FSW-BCSFB opening method could still provide a lower Racine’s scale, inflammation, oxidative stress, apoptosis, and zero mortality way to alleviate epilepsy [16].

**Fig. 1:**
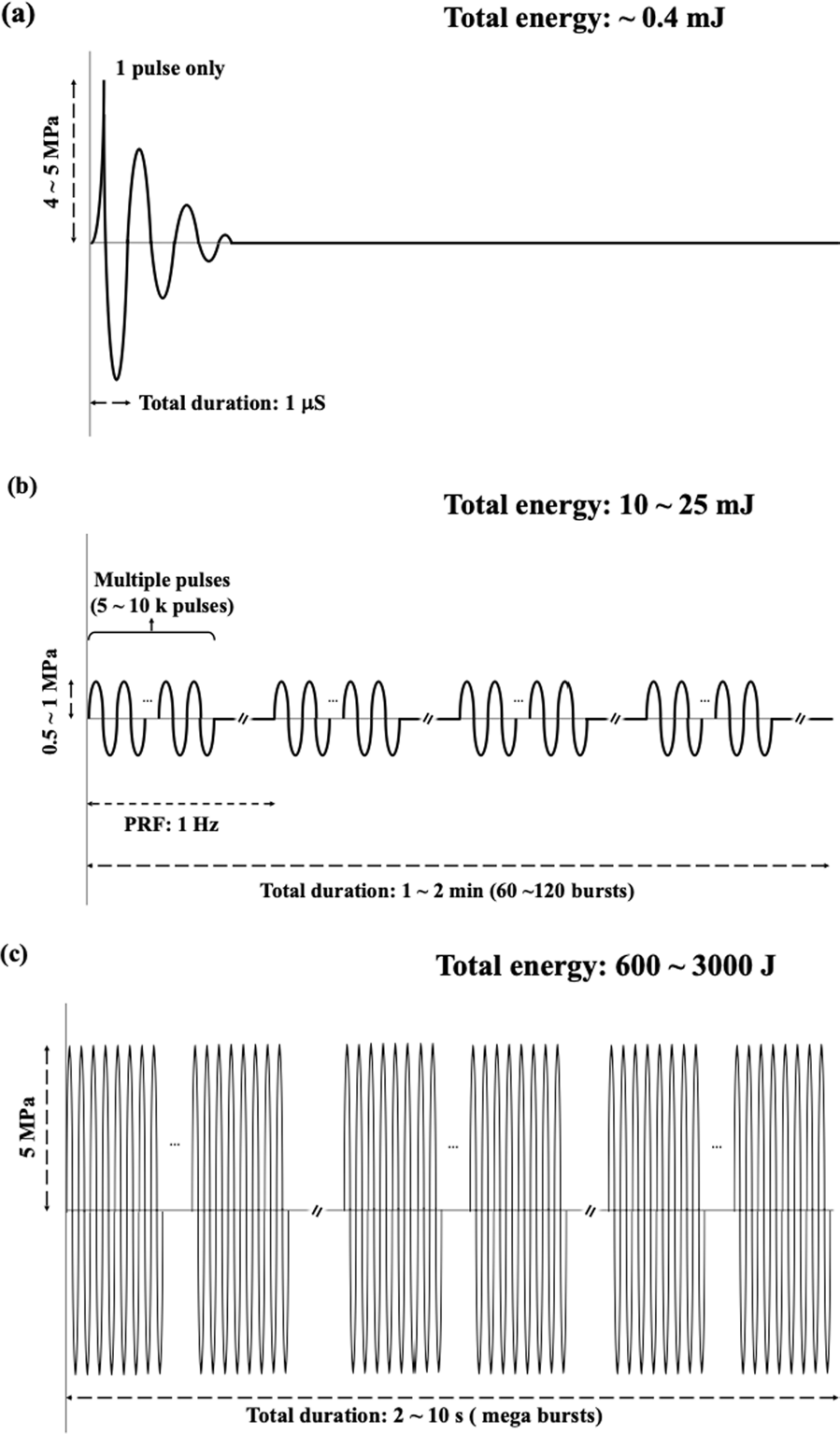
Comparisons of acoustic profile: **a** Single low-energy FSW pulse profile for BBB and BCSFB open; **b** HIFU pulses profile for BBB open; **c** HIFU pulses profile for tissue ablation

This study investigates the feasibility of applying a single low-energy extracorporeal focused shockwave pulse (FSW) with microbubbles to open the BCSFB for the delivery of large-molecule model drugs or therapeutics into CSF circulation. One of advantage for applying FSW is the lower total acoustic energy. Figure 1 shows the pulsing characteristics of the FSW device (Fig. 1a) used in the current study, the pulsed high-intensity focused ultrasound (HIFU) device, a preferred device to induce BBB opening (Fig. 1b), and the HIFU instrument to ablate tissue (Fig. 1c). Using the single low energy FSW pulse, the total energy required for BBB opening under the conditions shown in Fig. 1 is about 1/60,000 times that of by using pulsed HIFU. Moreover, the low energy FSW is only 1/7,000,000 of that for HIFU ablation. Furthermore, as FSW generates negligible heat, its application, exploiting acoustic related biological effects, would not be limited by tissue heating or related adverse effects [16–19].

In this study, we hypothesize that (i) FSW-based BCSFB opening will enhance drug passage; (ii) FSW exposure will induce BCSFB opening in specific brain regions with abundant choroid plexus (CP); and (iii) once drugs cross the BCSFB into the CSF circulation, they will remain long enough to maintain therapeutically sufficient drug concentrations. In this study, the location and efficiency of in vivo drug delivery were determined by measuring the fluorescence intensity of model drugs in the brain with an in vivo imaging system (IVIS) and by directly measuring the amounts of drugs in the CSF. Chemotherapeutic agents, at the same concentrations we achieved in the CSF, were tested for their ability to suppress the growth of selected CNS-targeted tumor cells in vitro. Finally, the feasibility and treatment efficacy of the FSW drug delivery technique were evaluated in a glioma rat model.

## Materials and methods

### Animals, materials and instruments

This study was approved by the ethics committee of the Laboratory Animal Center at the National Taiwan University College of Medicine (Approval No. 20170091 for the use of rats) and adhered to the experimental animal care guidelines. All rats (adult Sprague Dawley rats between 9 and 10 weeks of age) were obtained from the National Laboratory Animal Center (Taipei, Taiwan), fed a standard diet and housed in a temperature- and humidity-controlled room (19–23 °C; 40–70%, respectively) under a 12/12-h light–dark cycle. For more details, please refer to the supporting information.

### FSW-BCSFB opening and brain mapping

The commercial shockwave device and the FSW probe positioning platform were set up identically to those previously reported by Kung [19]. FSW with microbubbles was implemented following the established technique for single low-energy extracorporeal FSW pulses, which can minimize damage to the brain within a safe operating range [20]: single pulse (energy flux density 0.03 mJ/mm^2^) with SonoVue microbubbles (2 × 10^6^ MBs/kg).

**Fig. 2:**
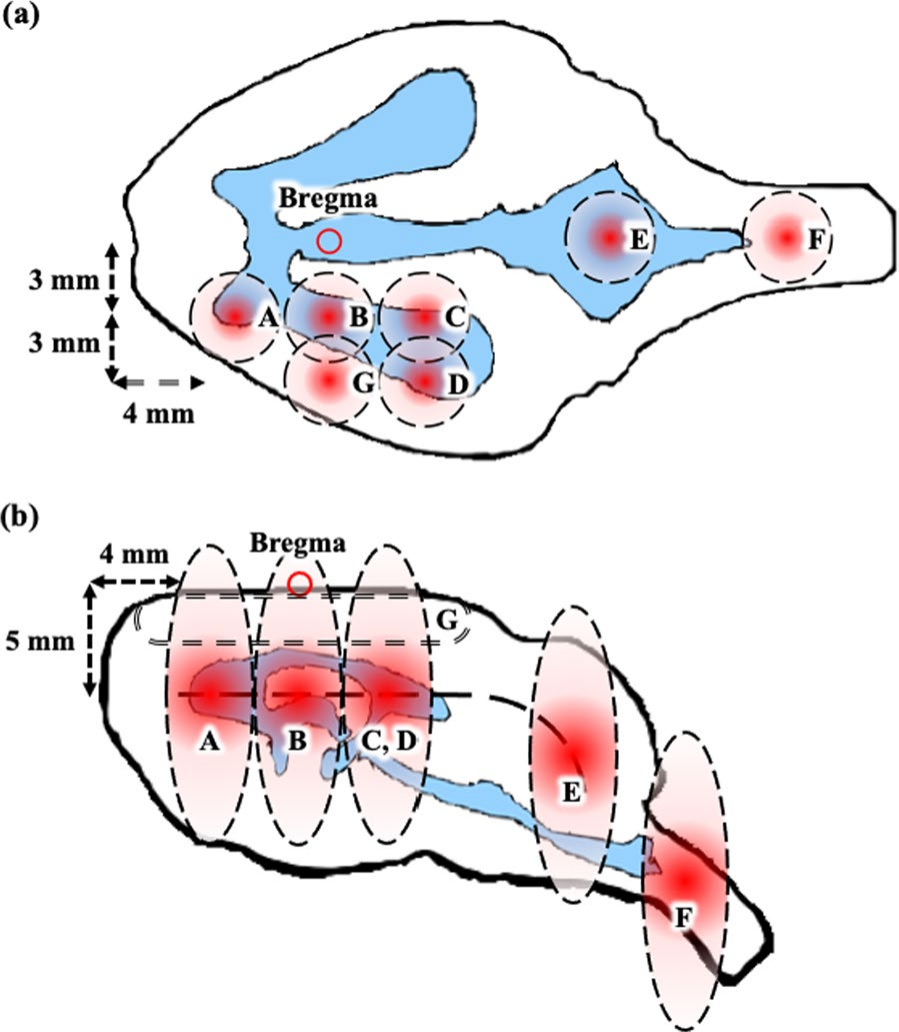
**a** Top view of FSW target positions on a rat brain. **b** Side view of FSW target positions on a rat brain. Positions A–C are respectively 4 mm, 0 mm, and − 4 mm anterior, and 3 mm lateral to the bregma. Position D is − 4 mm anterior and 6 mm lateral to the bregma. Positions E and F are respectively 12 mm and 16 mm posterior to the bregma. The depth of positions A–F and G relative to the surface of the brain are respectively 5 mm and 1 mm. The blue area corresponds to the ventricles in the brain. The focal dimensions (− 6 dB) of the shockwave at an intensity level of 0.1 (peak positive pressure 5.4 MPa; peak negative pressure − 4.2 MPa; energy flux density 0.03 mJ/mm^2^) are 3.8 mm in the *x*-axis, 3.8 mm in the *y*-axis, and 13.0 mm in the *z*-axis

To find the ideal location to open the BCSFB by FSW, we first selected different points in the rat brain where CP is abundant (Fig. 2a, b). The IVIS was then used to analyze the fluorescence signal distribution of the injected indicator for each brain slice to map good FSW treatment sites in the brain to improve experimental repeatability and accuracy.

**Fig. 3:**
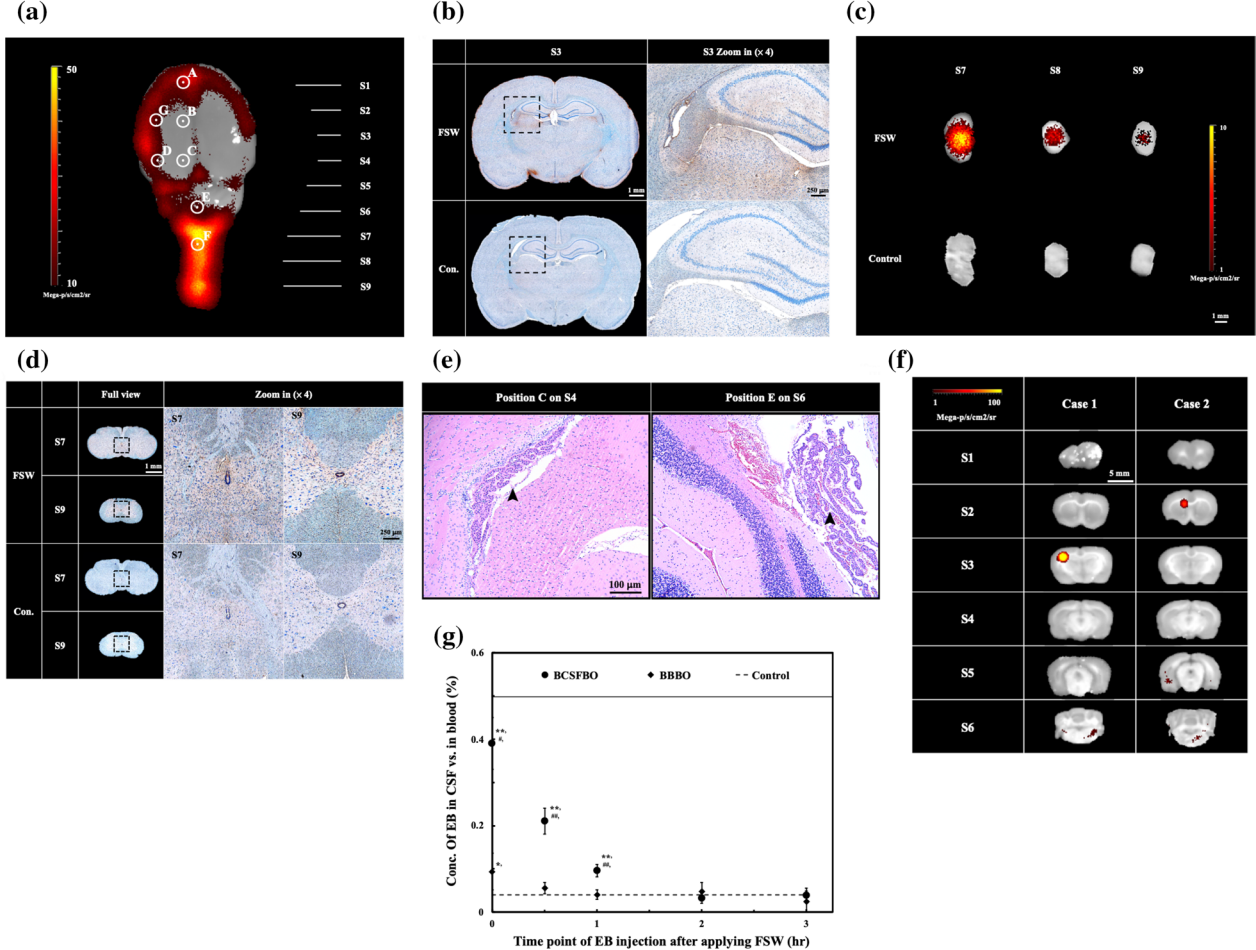
**a** Fluorescence images of a top view of a rat brain following FSW-induced BCSFB opening and the locations of the slices used for histological sectioning. **b** Histological sections at S3 stained with anti-FITC and hematoxylin for an animal treated with FSW at position C and an animal that did not receive FSW treatment (control, Con.). **c** Fluorescence images of histological sections (S7–S9) for the rat treated with FSW at position C and the control rat. **d** Histological sections at S7 and S9 stained with anti-FITC and hematoxylin for the animal treated with FSW at position C and the control animal. **e** H&E stains of position C (S4) and position E(S6). Black arrow: the choroid plexus. **f** Fluorescence images of histological sections S1–S6 after FSW treatment at position G. Fluorescence is exhibited locally since the FITC indicator cannot enter the CSF circulation**.** The sampling time was 3 h after FSW treatment. **g** Evans blue persistence in the CSF after FSW-BCSFB opening (FSW treatment at position C), FSW-BBB opening (FSW treatment at position G) or control treatment (no FSW treatment). The CSF sampling time is at 3.5 h after the FSW-treatment. *p, ^#^p < 0.05 vs. control and BBB opening, respectively; **p, ^##^p < 0.01 vs. control and BBB opening, respectively. In one-way ANOVA with the Tukey post hoc test (n = 5)

Once the rats were treated by FSW (single pulse, intensity level 0.1, peak negative pressure − 4.2 MPa; energy flux density 0.03 mJ/mm^2^) with microbubbles (2 × 10^6^ MBs/kg), 50 mg of either 70 kDa or 500 kDa FITC-dextran was administered via the tail vein. Three hours after FITC-dextran administration, the rats were sacrificed by a CO_2_ euthanasia instrument (followed the BU ASC guidelines for carbon dioxide euthanasia for rats and mice), and subsequent to a formaldehyde fixation process, their brains were sliced from locations S1 to S9 (shown in Fig. 3a) using brain matrices. The brain slices were then immediately placed into an IVIS scanning chamber to measure the fluorescence intensity of FITC-dextran, which was used to create a brain map for FSW-BCSFB opening. All IVIS data in the proposed research were analyzed using Living Image 3.1 (Caliper Life Sciences, Waltham, US).

### Duration of FSW-BCSFB opening and FSW-BBB opening

After determining the FSW triggering positions with a higher probability for FSW-BCSFB opening than FSW-BBB opening in subsection ‘FSW-BCSFB opening and brain mapping’, we evaluated the duration of FSW-UCA-induced BCSFB and BBB opening. Twenty rats each were treated with FSW-UCA at time 0 at positions suitable for opening the BCSFB (position C. on Fig. 3a) and BBB (position G on Fig. 3a). Then, 0.5 ml of 3% Evans blue was infused 0.5, 1, 2, and 3 h (with five rats at each time point for the two positions) after time 0. Then, samples of CSF were obtained 3.5 h after time 0, after which the rats were sacrificed. In addition, five rats were designated as the blank group and were not treated with FSW-UCA.

### Histopathologic sections and immunohistochemistry

To confirm the crossing of FITC-dextran across the BCSFB following the FSW treatments, after IVIS scanning, sliced rat brains were immersed in a 10% formaldehyde solution for 24 h. Subsequently, the sliced specimens were embedded in paraffin and subjected to immunohistochemistry to visualize FITC-dextran within the nuclei of the neurons. All 4-μm serial paraffin sections were deparaffinized by EZ prep (Ventana Medical Systems Inc., Tucson, AZ, USA). The slides were incubated with anti-FITC (FITC-11) at a 1:50 dilution for 30 min at 37 °C using the automated Ventana Benchmark XT (Ventana Medical Systems Inc.). Labeling was visualized with the UltraView DAB Detection Kit according to the manufacturer’s protocol. All sections were counterstained with hematoxylin in Ventana reagent. After staining, all slides were analyzed using a Ventana Dp200 slide scanner with the corresponding software, Ventana Image Viewer v3.2 Advanced.

### Duration of drug retention in the CSF circulation

To determine how long FITC-dextran can remain in the CSF circulation, rats were treated by FSW-UCA method and sacrificed 24, 72, and 120 h after FSW treatment, and brain slices were processed according to the procedure described in subsection ‘FSW-BCSFB opening and brain mapping’. The brain slices were then placed into the IVIS scanning chamber to measure FITC-dextran fluorescence intensity, i.e., the concentration distribution of the delivered drug in the CSF circulation.

### Efficiency of FSW-based drug delivery into the CSF

To evaluate the effectiveness of FSW in facilitating drug delivery into CSF circulation, the CSF concentrations of three indicators (Evans blue and 70 and 500 kDa FITC-dextran) and three clinical drugs (penicillin G, DOX, and BEV) were measured and compared before and after FSW treatment. Generally, these drugs have difficulty reaching the brain across the BBB or BCSFB under regular conditions [21]. Three hours after FSW-UCA treatment, samples of CSF were collected from the cisterna magna of the treated rats based on the protocols of a previous study [16, 22]. For more details, please refer to the Additional file 1.

### Efficiency of FSW-based delivery of drugs into the CSF with multiple FSW stimulations

To evaluate the possibility of applying multiple FSW stimulations to increase drug concentrations in the CSF, we applied FSW treatment at different positions or iterations under the following conditions: (i) C: a single FSW pulse at position C; (ii) C^10^: 10 FSW pulses at position C; (iii) A-B-C: a single FSW pulse each at positions A, B and C; and (iv) C^L^-C^R^: a single FSW pulse at position C for each hemisphere. The FSW pulses were administered at the same intensity level of 0.1 for all conditions. The indicator was 3% Evans blue (dissolved in 0.9% saline). Other conditions were identical to those listed in the previous subsections.

### Tumor cell viability

The procedures in the previous sections were used to determine the concentrations of both DOX and BEV in the CSF with and without FSW stimulation in vivo. Here, we investigated whether these concentrations of chemotherapeutic agents were sufficiently high to suppress the growth of three CNS-targeted tumor cell lines in vitro.

We used the different CSF concentrations of DOX and BEV obtained in the in vivo studies to C6, MDA, and MDA META cells in vitro and tested the viability of the cells using an alamarBlue assay. The advantages of alamarBlue include its technical simplicity, the absence of radioisotopes, its versatile detectability, the lack of a need to perform extraction, and its excellent reproducibility and sensitivity [26]. For more details, please refer to the Additional file 1.

### Application of the FSW-BCSFB opening technique in treating a glioma-bearing model

To evaluate the ability of our FSW-BCSFB opening technique to suppress tumor growth in vivo, the C6 glioma cell line was chosen to establish a brain tumor model. The rat C6 glioma cell line was transfected with the luciferase (LUC) gene and maintained following procedures described previously [31, 32]. The cells were then implanted in the rat brain according to the following modified procedure [25, 33]: (1) rats were anesthetized using 3% isoflurane in oxygen; (2) the caudo-putamen of each rat brain (0.5 mm anterior and 2.0 mm lateral to the bregma; 5 mm deep) was stereotactically injected with 10^5^ C6-LUC glioma cells in 5 µl using a Hamilton syringe within 15 min; and (3) the skull hole was sealed with bone wax, and the wound was rinsed with iodinated alcohol.

As shown in Fig. 8a, DOX (5 mg/kg) was infused through the tail vein with or without FSW treatment at position C on days 7, 10 and 13 following tumor cell injection using the procedures mentioned in subsection ‘FSW-BCSFB opening and brain mapping’. The rats were injected with 200 μl (100 mg/kg) of d-luciferin via the tail vein and then imaged by the IVIS to monitor treatment response (tumor size) on days 10, 13, 16 and 20 [32]. The following IVIS parameters were used: field of view B (6.5 × 6.5 cm), 1-min exposure time, medium binning, and f/stop = 1.

### Statistical analysis

Statistical analysis was performed using SPSS version 26. All data were expressed as mean ± standard deviation (SD) of at least five independent samples (N). In group comparisons, overall survival was calculated by the Kaplan–Meier method and the log-rank test was used to compare the survival curves. Other statistical evaluations were carried out with one-way ANOVA and post-hoc analysis (Tukey). A p-value of less than 0.05 was considered significant.

## Results

### FSW-BCSFB opening

We sought to assess the ability of the FSW technique to open the BCSFB by measuring the distribution of FITC-dextran through FSW stimulation at different points in the brain. We stained histological sections obtained at S3 with anti-FITC (DAB) and hematoxylin (blue) to evaluate rats that were and were not treated with an FSW pulse at position C and found a strong anti-FITC immunochromatographic reaction in areas around the third ventricle (Fig. 3b) and the gray matter around the central canal (Fig. 3d) only in the group treated with FSW pulses.

Figure 3c shows fluorescence images of histological sections S7–S9. After the BCSFB was opened by FSW, a strong fluorescence signal was observed around the center of the spinal cord in the FSW-UCA treatment group, but a similar signal was not observed in the control group. The immunoreactive sites depicted in the above images were all adjacent to regions of abundant CP and high CSF circulation. These results indicate that this FSW-based technique can indeed open the BCSFB and deliver drugs into the CSF circulation.

Figure 3e shows the H&E stains of position C (S4) and position E (S6). On position E, bleeding was found on cerebellum region 3 h after the FSW-treatment. However, no red cell extravasation, cell death or any hemorrhage were found on position C.

Additionally, Fig. 3f shows fluorescence images of each histological section after FSW treatment at position G for two different rats. The fluorescence is local since the FITC indicator cannot enter the CSF circulation.

Figure 3g illustrates the duration of BCSFB and BBB opening via evaluation of the concentration of an indicator, Evans blue, in sections ‘FSW-BCSFB opening and brain mapping’ and ‘Duration of FSW-BCSFB opening and FSW-BBB opening’ respectively following FSW treatment at position C and FSW treatment at position G, along with the concentration in a control animal (no FSW treatment). The concentration of Evans blue in the CSF was significantly higher following FSW-BCSFB opening than FSW-BBB opening. The concentration shown for the FSW-BCSFB technique corresponds to an opening duration of approximately 1–2 h. Besides, the mortality rate 3 h after FSW stimulation at positions E and F were respectively 60% and 70%, while that for the rest was 0%.

### Brain mapping by FSW delivery of FITC-dextran across the BCSFB

**Fig. 4:**
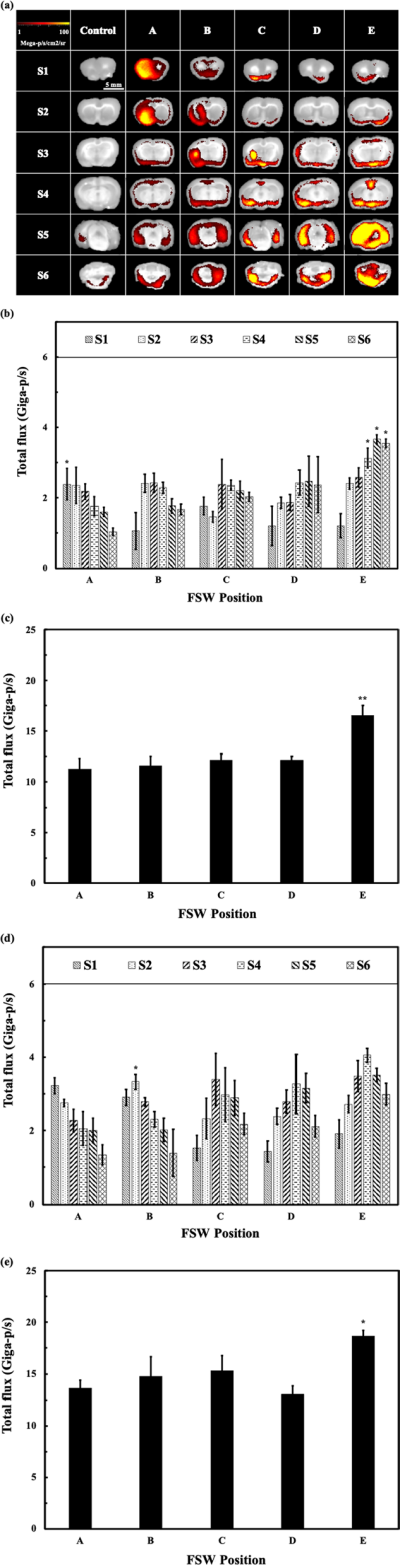
**a** Fluorescence images of each histological section from a rat after FSW treatment at different sites and from a control rat (no FSW treatment). The rats were injected with 70 kDa FITC-dextran as the indicator. The sampling time was 3 h after FSW treatment; **b** Quantitative results with 70 kDa FITC-dextran as the indicator for different brain sections following treatment at different sites. *p < 0.05 vs. other treatment positions, in the same section, in one-way ANOVA with the Tukey post hoc test (n = 5); **c** Total fluorescence intensity of (**b**) at different treatment sites. **p < 0.01 vs. other treatment positions, in one-way ANOVA with the Tukey post hoc test (n = 5); **d** Quantitative results with 500 kDa FITC-dextran as the indicator. *p < 0.05 vs. other treatment positions in the same section in one-way ANOVA with the Tukey post hoc test (n = 5); **e** Total fluorescence intensity of (**d**) at different treatment sites. *p < 0.05 vs. other treatment positions, in one-way ANOVA with the Tukey post hoc test (n = 5)

Figure 4a shows fluorescence images of brain sections S1–S6 at 3 h after FSW treatment at different positions (A–E) or after control treatment (no FSW treatment group). Figure 4b shows the normalized fluorescence intensity by two standard fluorescent cards for injections of 70 kDa FITC-dextran, while Fig. 4d shows the normalized fluorescence intensity by two standard fluorescent cards for injections of 500 kDa FITC-dextran. It can be clearly seen that the fluorescence intensities are higher in the brain regions and sections closer to the FSW stimulation areas, although the differences are not consistently significant between the two weights of FITC-dextran. For example, the total fluorescence flux of both 70 and 500 kDa FITC-dextran was higher at position B in brain slices S2 and/or S3 since position B is closer to the area captured by S2. The accumulated fluorescence intensities of all brain sections (Fig. 4c, e) were not significantly different except for that produced following treatment at position E.

Following stimulation of the left hemisphere by the FSW technique, the fluorescence signal for 70 kDa FITC-dextran could still be clearly seen in the contralateral hemisphere (Fig. 4a), indicating that the distribution was not limited to the stimulated area. This indicates that the proposed FSW treatment technique can open the BCSFB and deliver the indicator into the CSF circulation.

### Duration of indicator retention in the CSF circulation

**Fig. 5:**
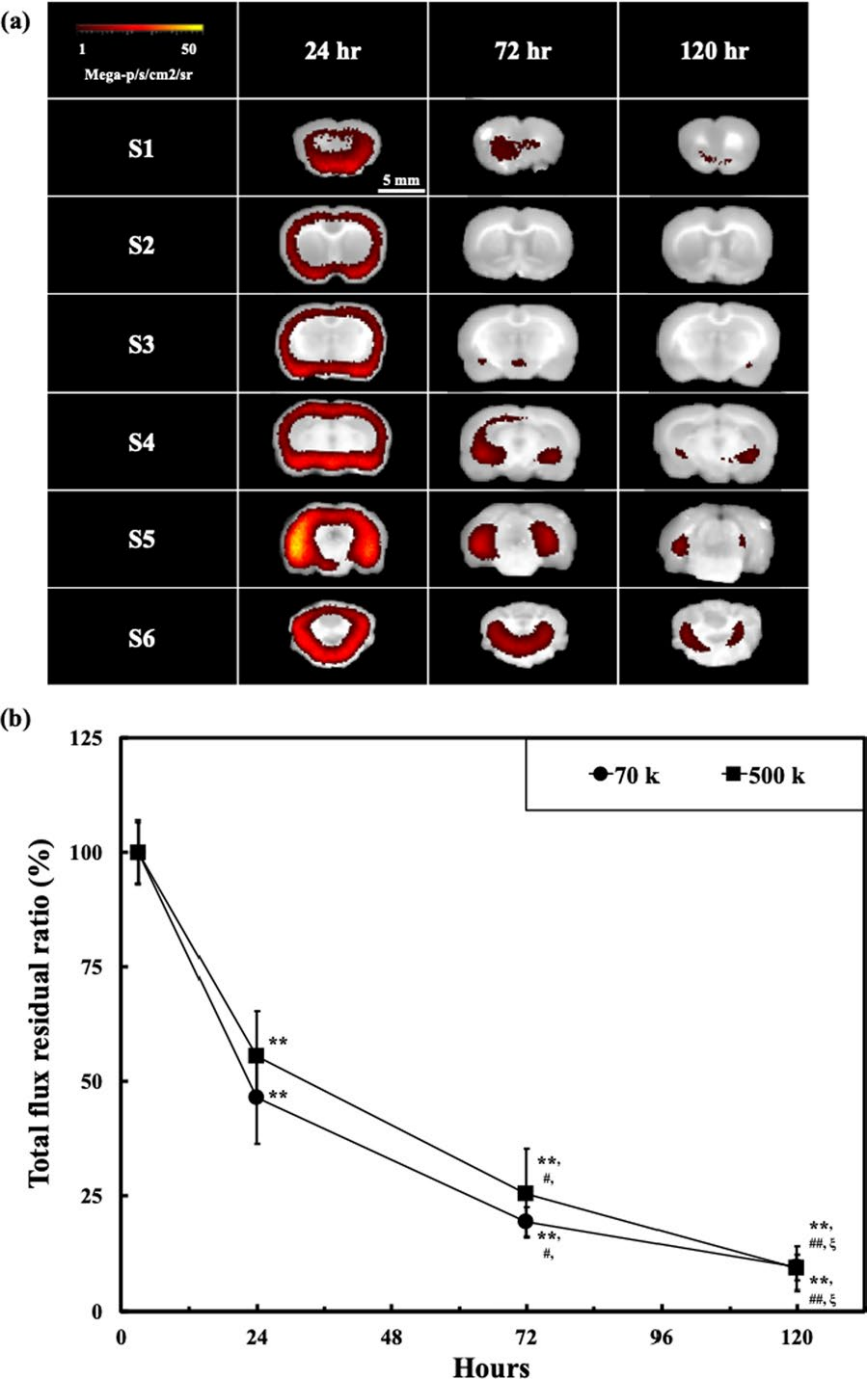
**a** Fluorescence images of different brain section levels at position C. The indicator was 70 kDa FITC-dextran. The sampling times was 24, 72 and 120 h after the FSW treatment. **b** Comparison of the average fluorescence flux across all sections using either 70 kDa or 500 kDa FITC-dextran as the indicator. The sampling times were 3, 24, 72, and 120 h after the FSW treatment at position C. ^#^p and ^ξ^p < 0.05 vs. 24 h and 72 h, respectively, sum of all sections; in one-way ANOVA with the Tukey post hoc test (n = 5); **p and ^##^p < 0.01 vs. 3 h and 24 h, respectively, sum of all sections; in one-way ANOVA with the Tukey post hoc test (n = 5)

Figure 5a shows fluorescence images for the brain histological sections harvested after 24, 72, and 120 h. The fluorescence intensity distributions were more uniform than those in Fig. 4a, and the strong signals around the stimulation points shown in Fig. 4a were absent. Figure 5b shows quantitative measurements of fluorescence intensity 3, 24, 72, and 120 h after FSW treatment. By normalizing the values by the intensity at 3 h, the intensity values at 24, 72, and 120 h were respectively 46.31%, 19.29%, and 9.38% with 70 kDa FITC-dextran and 55.40%, 25.42%, and 9.17% with 500 kDa FITC-dextran. Based on these results, the half-life of the fluorescence signal strength was approximately 24 h. Furthermore, significant differences in signal strength were seen only after 72 h. Therefore, this FSW-BCSFB opening provides a method to deliver drugs into the CSF circulation, with above 50% of drug concentrations at T0 (FSW-treatment) for around 24 h.

### Efficiency of FSW-based drug delivery into the CSF

Tables 1 and 2 compares CSF drug concentrations with and without FSW treatment. The numbers indicate the percentage of drug in CSF relative to that in the blood. As can be seen, the drug concentrations in the CSF following FSW treatment differed significantly from those in the CSF without FSW treatment (Table 1). These results bolster the findings made for FITC-dextran and illustrate that the FSW treatment technique is beneficial for different kinds of drugs.

**Table 1:** Drug concentrations in the cerebrospinal fluid

Drug	Molecular size (Da)	Drug concentration in the CSF (%)		
		FSW	Control	
Penicillin G	334	13.63 ± 4.51	2.62 ± 1.38	**
Doxorubicin	544	59.09 ± 11.18	6.92 ± 4.67	**
Evans blue [C]	67,000	0.39 ± 0.06	0.04 ± 0.04	**
FITC-Dextran	70,000	1.41 ± 0.37	0.16 ± 0.18	**
Bevacizumab	149,000	33.34 ± 10.44	14.6 ± 5.79	*
FITC-Dextran	500,000	2.44 ± 0.78	0.11 ± 0.39	*

* and **, p < 0.05 and 0.01, respectively, vs. control, in one-way ANOVA with the Tukey post hoc test (n = 5)

Sampling time: at 3 h after the FSW-treatment; FSW-position: C

Unit: %, CSF/blood drug concentration

**Table 2:** EB concentrations on cerebrospinal fluid with multiple stimulation strategy

Multiple stimulation strategy		Drug concentration in the CSF (%)		
Locations	Pulse(s)	FSW	Control	
C	1	0.39 ± 0.06	0.04 ± 0.04	**
C	10	5.58 ± 2.23		**
A-B-C	1	1.28 ± 0.53		**
C^L^-C^R^	1	1.73 ± 0.47		**

**, p < 0.01 vs. control, in one-way ANOVA with the Tukey post hoc test (n = 5)

Sampling time: at 3 h after the FSW-treatment

Unit: %, CSF/blood Evans blue concentration

**Fig. 6:**
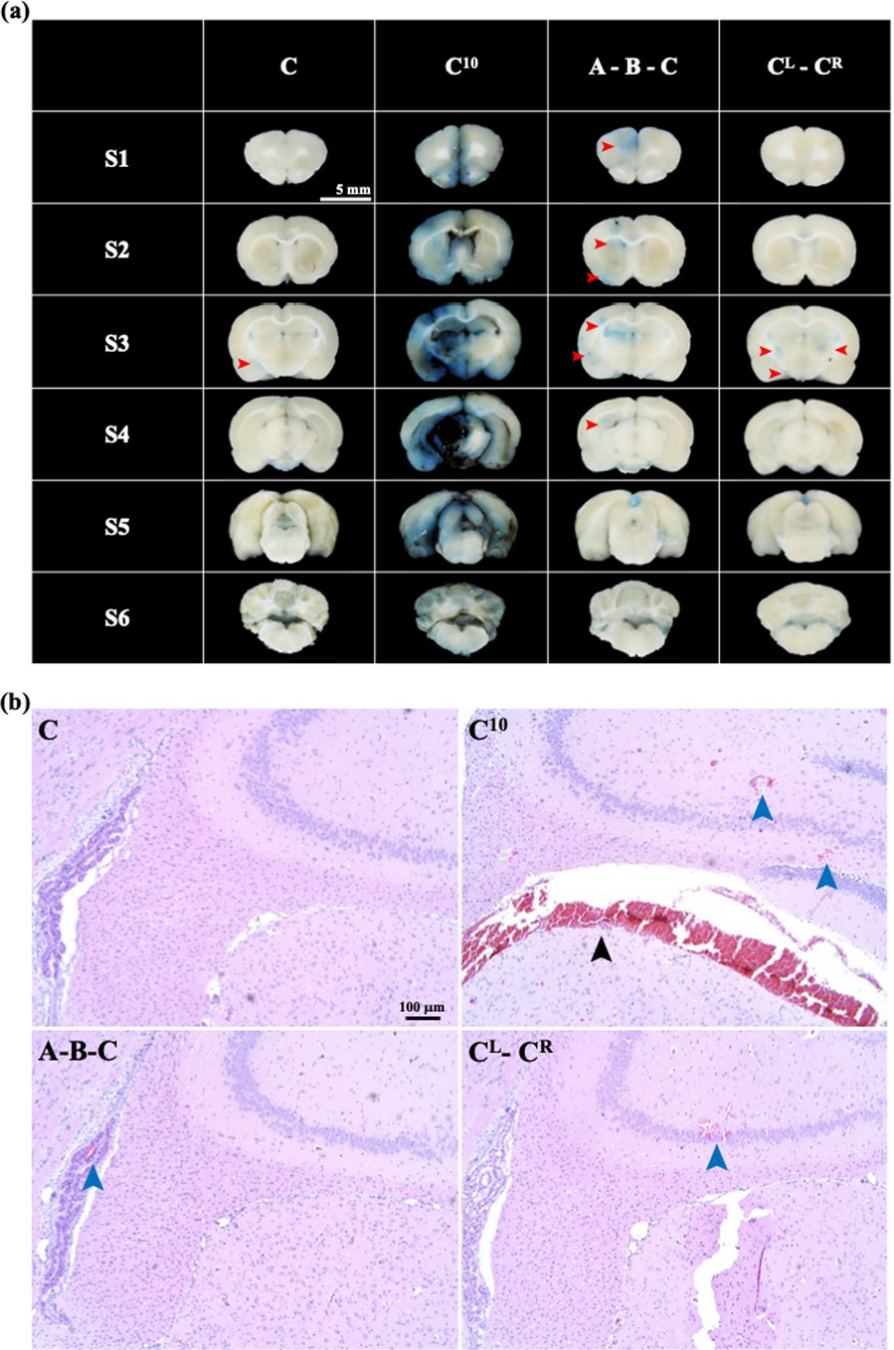
**a** Histological sections and **b** H&E stains on S3 after applying the FSW technique at different positions and for different iterations. Red arrows: the BCSFB and BBB opening regions; Blue arrow: the red blood cell extravasations; Black arrow: the bleeding; C: single FSW pulse at position C; C10: 10 FSW pulses at position C; A-B-C: single FSW pulses at positions A, B and C; CL-CR: a single FSW pulse at position C on both hemispheres. The shockwave pulses were administered at an intensity level of 0.1. The indicator was 3% Evans blue (predissolved in 0.9% saline). The scale bar on (**a**) and (**b**) respectively represents 5 mm and 100 μm (n = 5)

Figure 6a shows histological sections after the application of FSW at different positions and iterations. In addition, the BCSFB and BBB opening regions (the blue-stained areas), indicated by red arrows, are at similar locations to the high FLUX-LUX intensity locations at different FSW positions in Fig. 4a. Figure 6a shows H & E stains on S4 of Fig. 6a, in which, the blue arrows indicate the locations of the red blood cell extravasations (on A-B-C and CL-CR groups), and the black arrow indicate the bleeding (in C10 group). Unfortunately, the brain bleeding rat was dead within 3 h after the FSW-treatment. In the current study, the mortalities of the C, C10, A-B-C, and CL-CR groups were respectively 0%, 20%, 0%, and 0% (Table 2). Despite these groups showing a 3- to 4.5-fold increase in CSF drug concentration (Table 2), a single shockwave pulse is still recommended for BCSFB opening to avoid the unexpected brain bleeding.

### Tumor cell viability

**Fig. 7:**
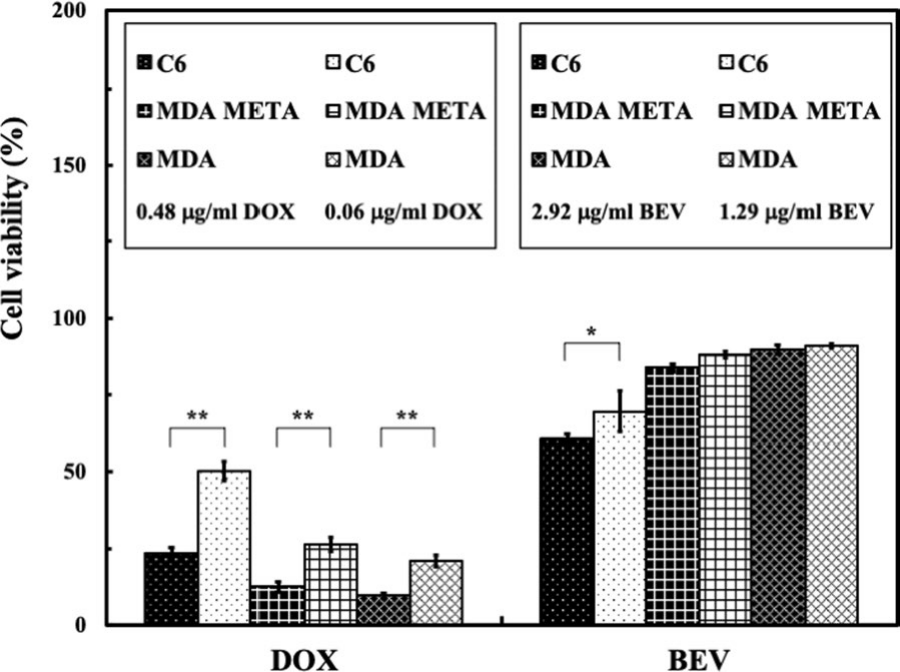
Cell viability of C6, MDA META, and MDA cells cultured with DOX or BEV after 24 h. *p < 0.05 and **p < 0.01, in one-way ANOVA with the Tukey post hoc test (n = 5)

Figure 7 shows the viability of C6, MDA META, and MDA cells cultured with DOX or BEV at the in vivo CSF concentration of each drug corresponding to rats with (FSW) or without (Con) shockwave treatment. For the cells cultured with DOX, the viabilities in the FSW group were significantly different from those in the control group for all three cell line types. For the cells cultured with BEV, the cell viability of the FSW group was significantly different from that of the control group for only the C6 cell line. Furthermore, at the studied concentration, DOX showed good tumor cell suppression in all three cell lines.

### Application in treating a glioma-bearing model

**Fig. 8:**
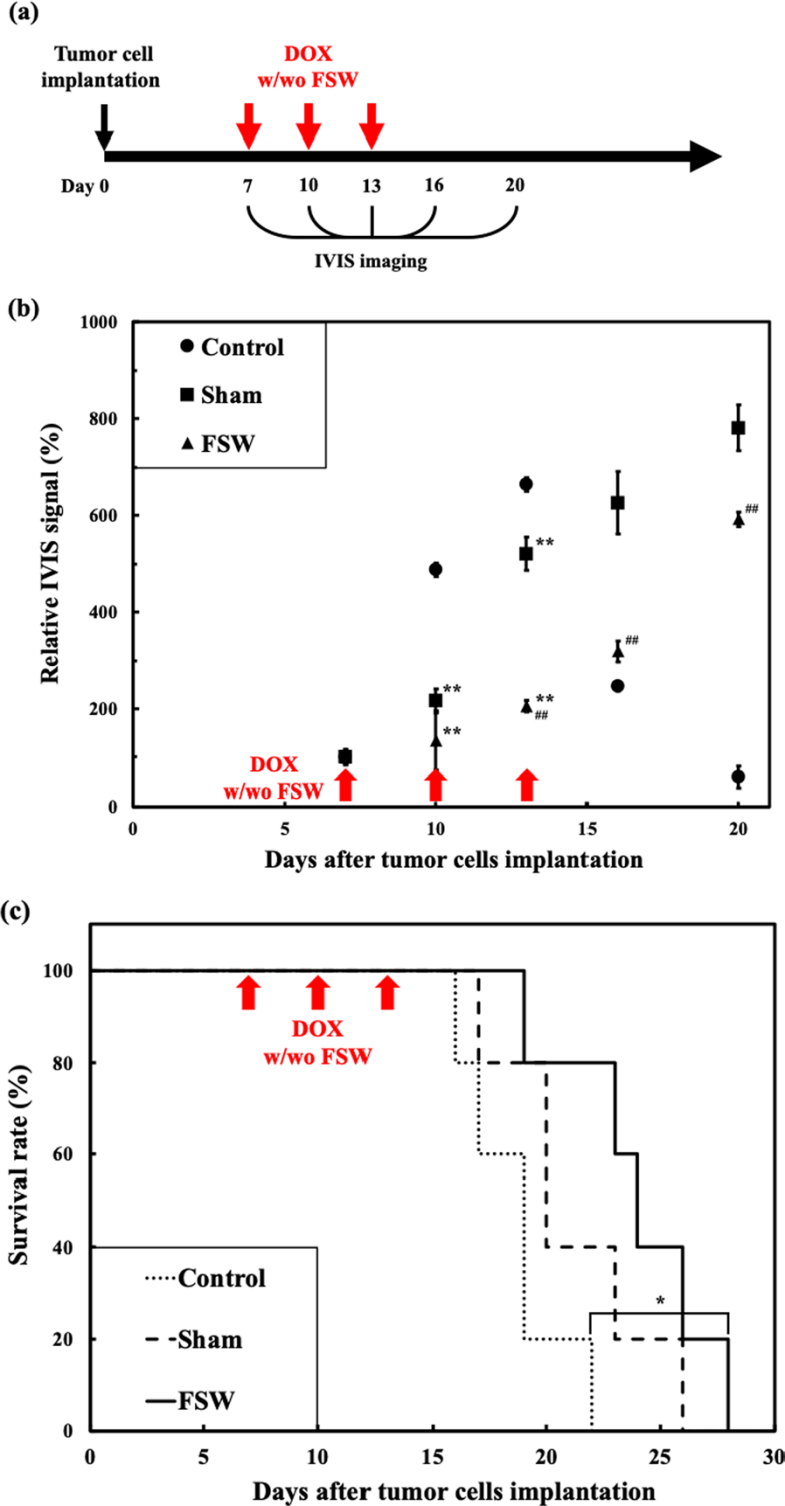
**a** Experimental timeline for doxorubicin and FSW treatment. **b** Longitudinal brain tumor monitoring via luciferase activity using an IVIS, wherein the relative IVIS signal (%) is the IVIS signal intensity at the specified sampling day divided by the IVIS signal intensity 7 days after tumor cell implantation. **p and ^##^p < 0.05 vs. control and Sham, respectively, in one-way ANOVA with the Tukey post hoc test (n = 5). **c** Comparison of animal survival duration among the FSW group (FSW + DOX), Sham group, and control group (*p < 0.05 in one-way ANOVA with the Tukey post hoc test). In which, Control: GBM-rats without any treatment; Sham: GBM-rats with DOX treatment only; FSW: GBM-rats with FSW-DOX treatment (n = 5)

Figure 8b shows brain tumor (C6 cells) growth following DOX injection monitored by measuring luciferase activity using the IVIS imaging system. The FSW and control groups showed major differences in fluorescence intensity after the first treatment. However, the FSW and Sham groups started to show significant differences after the second treatment. Figure 8c shows an analysis of animal survival via Kaplan–Meier curves. Compared with the control group, the FSW group, but not the Sham group, showed a significant difference in survival (*p < 0.05). However, the difference between the Sham and FSW groups was not significant. This indicates that the proposed FSW-BCSFB opening method can significantly improve the antitumor efficacy of DOX in the glioblastoma model.

## Discussion

The BCSFB is regarded as the gatekeeper of blood circulation into the CSF space. By stimulating specific areas of the brain using a single FSW pulse with microbubbles, we not only temporarily opened the BCSFB to facilitate the delivery of therapeutics into the brain via the CSF circulation, but also achieved therapeutic concentrations of DOX that suppressed the growth of glioblastoma multiforme tumors in an in vivo model. Our results indicate that this technique has potential for treating various CNS disorders by opening the BCSFB and increasing drug concentrations in the CSF circulation.

The BCSFB is formed by epithelial cells of the CP located primarily in the four ventricles of the brain. The CP epithelium, which forms the BCSFB, is a unique single layer of epithelial cells situated at the interface between the blood and CSF. In contrast to the structures of the BBB, the BCSFB is formed by tight junctions between CP epithelial cells instead of endothelial cells, restricting the movement of molecules leaking from fenestrated capillaries into the CSF. By analyzing the distal diffusion of fluorescence to different parts of the brain and spinal cord, our study demonstrated a site-specific strategy for opening the BCSFB by FSW. We found that by stimulating the rat brain at different positions with the same FSW intensity, the BCSFB could be opened in positions near the lateral ventricles (positions A–D), areas with abundant CP (Fig. 3e), but not at the brain parenchyma (position G) (Fig. 3f). At this moment, we still can not obtain high-power enough pictures and evidences of FSW-disrupted choroid plexus. The permeable choroid plexus may be caused by whether the acoustic-pressure disruption or tight junction opening such as TRPV4 channel or other mechanism [34].

Additionally, the fluorescence following stimulation at position G was found only locally, suggesting that only the BBB was opened, not the BCSFB. The comparison of indicator concentrations in the CSF (Fig. 3g) provided additional evidence of the high correlation between FSW position and BCSFB opening.

Conventionally, as compared with ultrasound, shockwave is believed to have higher potential to tissue damages while applying on brains. However, as shown in our previous study, the fine-tuned single low energy FSW pulse shows improved control of cavitation without detectable hemorrhage or apoptosis, and a lower inflammation, apoptosis, and free radical generation [reactive oxygen species (ROS) and reactive nitrogen species (RNS)] in the brain [16, 19]. The single low energy FSW pulse may provide an alternative way to deliver drugs into brains, and may benefit future neuro-oncology and neuropharmacology research and applications.

Common methods of FSW- and HIFU-based BBB opening provide a time window of 1 h or less and deliver drugs to only a thin region (approximately 2 mm^3^) of the rat brain. Even with a 72-h time window, HIFU-BBB opening can deliver drugs to only approximately 30 mm^3^ of the rat brain [16, 20, 35]. In contrast, by inducing BCSFB opening in suitable positions, our FSW-BCSFB opening technique could provide not only a method to deliver drugs into the CSF circulation (nearly the whole rat brain and spinal cord) but also provides a 1- to 2-h time window for drug administration.

In this study, the operating parameters of the FSW-BCSFB opening technique were identical to those of the FSW-induced BBB opening technique reported in our previous study [16, 20]. However, the FSW stimulation positions were different. In our previous research, the FSW focus was intentionally positioned 5 mm beneath the scalp surface, 3 mm caudal, and 3 mm right or left of the bregma of the rat skull [16, 19, 20]. Thus, the maximal pressure area was positioned at the cortex of the rat brain for BBB opening. Here, the positions for FSW-BCSFB stimulation are beside the blue area in Fig. 2, especially near the CP-rich area. Limited by the need to control the intensity level and energy of the FSW pulses, the precise threshold for FSW-BCSFB opening is unknown. However, the data obtained in this study supports the concept that FSW can be used to selectively manipulate the permeability of the BBB and BCSFB.

It is worth noting that stimulation at areas close to the fragile cerebellum and brain stem area (E and F) may result in mortality. The mortality rate 3 h after FSW stimulation at positions E and F were respectively 60% and 70%, while that for the rest was 0%, probably because positions E and F are located near the cerebellum and brain stem, which are essential to many vital functions [36].

CSF circulates in the ventricles and the subarachnoid space, and plays an essential role in CNS homeostasis, including protection, waste clearance, and nutrient transport. Recent studies have demonstrated that a back-and-forth movement of the CSF is produced by respiratory and cardiac pulsations surrounding small penetrating arteries, providing exchanges between the CSF and interstitial fluid of the brain parenchyma. The pathway consists of a para-arterial influx route for CSF to enter the brain parenchyma [37]. Moreover, drugs delivered into the CSF can diffuse into the brain parenchyma and contact brain cells. A recent study demonstrated the possibility of using focused ultrasound to facilitate the CSF drainage through the glymphatic pathway from periarterial to perivenous CSF spaces [38, 39]. The technique shown in this study not only enhances the transport of drugs across the BCSFB and the BBB but also has the potential to push drugs further through ventricle and glymphatic pathways to brain cells due to the pulsating nature of the mechanical waves.

Several CNS disorders have been found to spread via the subarachnoid spaces and CSF. Among them, leptomeningeal carcinomatosis (LC), consisting of tumor cells that metastasize throughout the CSF space to the CNS, is the most devastating and terminal type and is frequently seen in lung cancer and breast cancer patients [40]. Intrathecal administration of antitumor agents such as trastuzumab, which has poor CNS penetration following intravascular infusion, has shown efficacy in patients with HER2-positive breast cancer with LC [41]. Our technique may provide a less invasive alternative for the treatment of LC; through the use of a multiple stimulation strategy (for example, the A-B-C or the C^L^-C^R^ strategy shown in Fig. 6), the concentration of treatment drugs could be controlled.

Comparing the accumulated fluorescence intensity (Fig. 4c, e) of both 70 and 500 kDa FITC-dextran, no significant difference was observed after FSW stimulation at positions A–D. This may be due to the wide distribution of the CSF in the CNS. Once a drug enters the CSF circulation, it can circulate in the whole CSF system, and its half-life is longer than that in the blood circulation [42]. Stimulation at position E produced significantly higher concentrations of FITC fluorescence, but the increased likelihood of mortality makes it a poor choice for FSW treatment.

The molecules sucrose (360 Da) and inulin (5 kDa) slowly cross the CP to enter the CSF with a CSF/blood ratio (CBR) of approximately 10% [43], and the protein transthyretin (55 kDa) can cross the CSF barrier with a 5.6% CBR. However, other molecules (> 55 kDa), such as albumin (67 kDa), IgG (150 kDa), and IgM (900 kDa), have respective CSF/blood ratios of only 0.49%, 0.22% and 0.03% [44, 45]. Besides, as shown in Fig. 4, the FSW-BCSFB opening technique can enhance the BCSFB permeability to even large molecules to deliver drugs into the CSF circulation. Furthermore, regardless of the FSW stimulation position, once the BCSFB is opened, the indicator is distributed in the CSF circulation from the brain ventricles to the spinal cord. This method can also provide a one-day half-life of drug delivery, as shown in Fig. 5b. Therefore, this method could not only allow the delivery of molecules ten-times larger than normally allowed through the BCSFB channel for future drug delivery, but also keep drugs in the CSF circulation for at least one day.

In this study, the animal species is SD rat. The species differences in brain size will bring concerns such as the relative distance from CSF to deep brain structures in rats and humans. However, based on previous studies, those brains on the higher-level species are with higher folding brain structure. Once, the drugs are delivered into the ventricles, the subarachnoid space, or the parenchyma, they will be much easier to be delivered into the whole brain since the glymphatic pathways in the gyrencephalic brain [46, 47]. Moreover, for the current FSW technique, the penetration depth is adjustable from 0.5 cm to around 15.0 cm using different gel pads according to actual needs.

The in vivo DOX concentrations in the CSF following FSW treatment showed a good suppressive ability against all three cancer cell lines tested in vitro (Fig. 7), C6, MDA META, and MDA, probably due to the interaction of DOX with tumor cell DNA by direct intercalation and inhibition of macromolecular biosynthesis. In contrast, the suppressive effect of BEV was poor, since BEV can exert its effect only by inhibiting vascular endothelial growth factor A (VEGF-A) and angiogenesis. Under the in vitro experimental mechanism described here, BEV was unable to exert its antiangiogenic ability [48, 49]. Furthermore, the FSW-BCSFB opening technique combined with DOX shows potential in the suppression of both orthotopic and metastatic brain tumors.

The results in Fig. 8 suggest that our FSW technique can be combined with DOX to suppress glioblastoma tumors in a rat model. Significant tumor suppression could be seen on day 13 or after the 2nd FSW treatment. Significant survival improvement was also seen between the FSW and control groups. There was no significant survival improvement between the FSW and sham groups, which may be caused from the limited rat number. Referred to the Fig. 8b, that already showed the significant improvement on the GBM cell growth rate between the FSW and sham groups. On the other hand, in Fig. 8b, the reason why the signal of the control group dropped after day 13 is probably due to excessive tumor size and internal necrosis when the GBM cells grew too fast to obtain enough nutrition [50].

## Conclusions

This is the first study to demonstrate that the BCSFB can be noninvasively opened in selected brain regions by a single FSW pulse with microbubbles. Though we cannot perfectly avoid BBB opening, we successfully show that the positions of FSW stimulation, i.e., near choroid plexus, enhance the probability of BCSFB opening. BCSFB opening allowed significant increase in the concentrations of certain CNS-impermeable indicators as well as medications such as penicillin G, DOX, and BEV into the CSF, brain and spinal cord region. Moreover, the increased CSF concentrations of DOX were found to be effective for the in vitro suppression of glioblastoma multiforme (C6), breast tumor (MDA) and MDA brain metastasis (MDA META) cells. Furthermore, FSW-treated glioblastoma bearing animals displayed significantly longer survival than untreated animals. These results suggest that FSW treatment could be an alternative way to combat CNS disorders by opening the BCSFB and enhancing the therapeutic efficiency of medication through entry into the CSF circulation. This innovative approach may benefit future treatments for neurodegenerative disorders, CNS infection, brain tumors, and leptomeningeal carcinomatosis.

## Supplementary Information

**Additional file 1.** Material and method details.

## Data Availability

The datasets analyzed during the current study are available from the corresponding author on request.

## Abbreviations

BBB: Blood–brain barrier
BCSFB: Blood-cerebrospinal fluid barrier
BEV: Bevacizumab
C6: Glioblastoma multiforme cells
DOX: Doxorubicin
FSW: Focused shockwave pulse
GBM: Glioblastoma multiforme
IVIS: In vivo imaging system
LUC: Luciferase
MDA: Breast tumor cells
MDA META: MDA brain metastasis cells
UCA: Ultrasound Contrast Agents
