# Supplementary material for: Facilitating drug delivery in the central nervous system by opening the blood-cerebrospinal fluid barrier with a single low energy shockwave pulse
Source: Fluids Barriers CNS. 2022 Jan 6;19:3. doi: 10.1186/s12987-021-00303-x (PMC8740485; doi:10.1186/s12987-021-00303-x)
Supplement: Supplementary file 1 — Additional file 1. Material and method details. [file 12987_2021_303_MOESM1_ESM.docx]

**Facilitating drug delivery in the central nervous system by opening the blood-cerebrospinal fluid barrier with a single low energy shockwave pulse**

Yi Kung^a^, Kuan-Yu Chen^b^, Wei-Hao Liao^a^, Yi-Hua Hsu^c^, Chueh-Hung Wu^a^, Ming-Yen Hsiao^a^, Abel P.-H. Huang^c*^, Wen-Shiang Chen^a, d*^

^a^Department of Physical Medicine and Rehabilitation, National Taiwan University Hospital & National Taiwan University College of Medicine, Taipei City, Taiwan.

^b^Division of Pulmonology, Department of Internal Medicine, National Taiwan University Hospital and College of Medicine, Taipei City, Taiwan.

^c^Department of Surgery, National Taiwan University Hospital, Taipei City, Taiwan.

^d^Institute of Biomedical Engineering and Nanomedicine, National Health Research Institutes, Miaoli, Taiwan.

*Equally contributing corresponding authors

Abel P.-H. Huang, E-mail: [how.how0622@gmail.com](mailto:how.how0622@gmail.com)

Wen-Shiang Chen, E-mail: [wenshiang@gmail.com](mailto:wenshiang@gmail.com)

**Additional materials**

**2. Materials and methods**

**2.1 Animals, materials and instruments**

Table 1s shows the Summary of all the groups. Isotonic sodium chloride solution (0.9 %) was provided by Taiwan Biotech Co., Ltd. (Taoyuan, Taiwan) and was sterile filtered through a 0.22 μm polyethersulfone (PES) membrane (Millipore syringe filter) from Polyplus-transfection (Illkirch, France). Fluorescein isothiocyanate-dextran (FITC-dextran; molecular weights: 70 kDa and 500 kDa), penicillin G (molecular weight: 334 Da), hematoxylin, and Evans blue were purchased from Sigma-Aldrich, Inc. (Missouri, USA). When administered into the circulation, Evans blue immediately binds to serum albumin and forms a 67 kDa molecular complex. Doxorubicin (DOX, molecular weight: 579.98 Da) was purchased from Pharmacia & Upjohn (New York, USA). Bevacizumab (BEV, Avastin®; molecular weight: 579.98 Da) was purchased from F. Hoffmann-La Roche (Basel, Switzerland). BEV Pharmacokinetic and Rat Penicillin ELISA Kits were purchased from MyBioSource (San Diego, USA).

Firefly D-luciferin potassium salt was purchased from Biosynth AG (Lake Constance, Switzerland). Anti-FITC (FITC-11) was purchased from Santa Cruz Biotechnology (CA, US). An UltraView DAB detection kit was acquired from Ventana Medical Systems (AZ, USA). Ultrasound coupling gel (CG955, sonic resistance: 1.55 ± 0.05 Mrayl, pH 7.0 ± 0.05) was obtained from Ceyotek (Chiayi City, Taiwan).

An FSW device (PiezoWave) was purchased from Richard Wolf (Knittlingen, Germany). The ultrasound contrast agents (UCAs), SonoVue microbubbles, were acquired from Diagnostics (Milan, Italy). The Laboratory Animal Center at the National Taiwan University College of Medicine was equipped with an IVIS and an XGI-8 gas anesthesia system (PerkinElmer, Waltham, US). A slide scanner (Ventana Dp200) and its software (Ventana Image Viewer v3.2) were obtained from F. Hoffmann-La Roche (Basel, Switzerland). A microplate reader (Infinite 2000 Pro) and its software (i-control) were procured from Tecan Austria (Grodig, Austria).


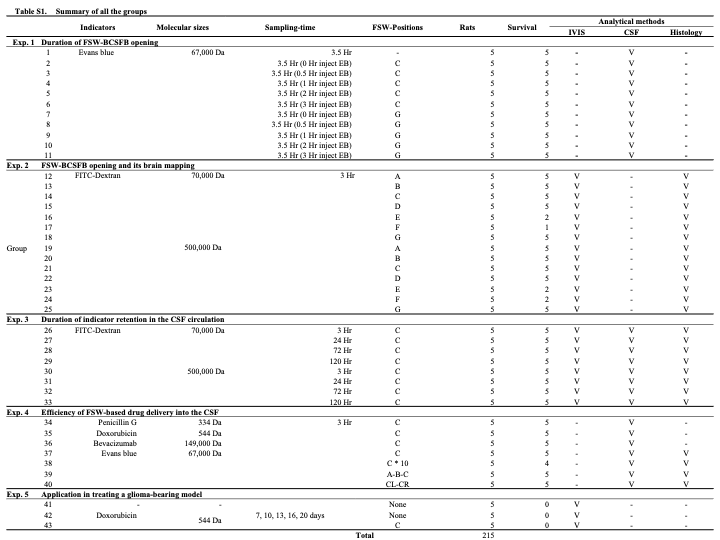


**2.6 Efficiency of FSW-based drug delivery into the CSF**

Evans blue was evaluated by measuring the absorbance at 620 nm, while FITC-dextran was evaluated by measuring the fluorescence at λex 493 nm and λem 520 nm. Penicillin G is an important clinical drug for treating pathogens that cause CNS infections. Due to its low CNS crossing capacity, a high therapeutic dose is needed during the treatment period, but high concentrations of penicillin G may induce serious side effects such as leukopenia, thrombocytopenia, seizure, and heart failure [23]. The penicillin G concentration was measured with a Rat Penicillin ELISA kit (MBS3808772, MyBioSource, California, USA).

Two other chemotherapeutic agents, DOX and BEV, were also evaluated for BCSFB penetration after FSW stimulation. Despite its poor CNS biodiversity, DOX is a common drug for breast cancer, bladder cancer, and lymphoma therapies [24]. BEV is an antiangiogenic monoclonal antibody that is commonly used in glioblastoma treatment, as well as colon cancer, lung cancer, and renal-cell carcinoma therapies. BEV overdose may induce hemorrhage, gastrointestinal perforation, and heart failure [25]. The concentration of DOX was determined from its intrinsic fluorescence (λex 490 nm and λem 560 nm), while the concentration of BEV was determined by a BEV pharmacokinetic ELISA kit (MBS378012, MyBioSource, California, USA).

**2.8 Tumor cell viability**

The C6 cell line is a standard cell line used to establish glioblastoma multiforme (GBM) models, whose infiltration into the CNS leads to neurological dysfunction and eventually death [27]. The MDA cell line (MDA-MB-231) is a highly aggressive, invasive and poorly differentiated triple-negative breast cancer [28]. Brain metastases are among the most feared complications in breast cancer and are traditionally managed to a limited degree by chemotherapy or targeted therapy. In addition, cancer cells that metastasize to the brain are different from those of the primary tumor [29].

To provide more information on drug treatments for brain metastases, a human breast cancer cell model for brain metastasis was chosen. The brain-seeking clone (MDA META) exclusively spreads to the brain at a histological level after six repeated passages in brain metastases and in culture. To prepare the MDA META cell line, parental MDA-MB-231 breast cancer cells were inoculated into the heart of host rats (4 weeks old) and spread to the brain approximately 3-4 weeks after inoculation. The MDA-MB-231 cells in the brain metastases were isolated, grown in culture as (MDA-MB-231 P1) and then reinoculated into the heart. The MDA-MB-231 P1 cells that metastasized to the brain were then isolated and grown in culture (MDA-MB-231 P2) again. This procedure was repeated six times. MDA-MB-231 P6 cells were then used as the brain-seeking clone (MDA META).

C6, MDA, and MDA META cells were seeded at 10,000 cells per well in a 96-well plate and then treated with DOX or BEV one day after plating. Cell viability following drug treatment was assessed 5 days later using the alamarBlue assay (Invitrogen) according to the manufacturer’s guidelines and previous work [30]. All culture experiments were performed in triplicate with a minimum of 5 wells per condition.
